# Convergent HIV-1 Evolution upon Targeted Destabilization of the gp120-gp41 Interface

**DOI:** 10.1128/JVI.00532-21

**Published:** 2021-11-23

**Authors:** Alba Torrents de la Peña, Iván del Moral Sánchez, Judith A. Burger, Ilja Bontjer, Gözde Isik, Dirk Eggink, Marit J. van Gils, Rogier W. Sanders

**Affiliations:** a Department of Medical Microbiology, Academic Medical Center, University of Amsterdam, Amsterdam, The Netherlands; b Department of Microbiology and Immunology, Weill Medical College of Cornell University, New York, New York, USA; c Department of Integrative Structure and Computational Biology, The Scripps Research Institute, La Jolla, California, USA; Emory University

**Keywords:** HIV, HIV vaccine, envelope glycoprotein, evolution

## Abstract

The HIV-1 envelope glycoprotein (Env) trimer is responsible for viral entry into target cells and is the sole target of neutralizing antibodies. The Env protein is therefore the focus of HIV-1 vaccine design. Env consists of two noncovalently linked subunits (gp120 and gp41) that form a trimer of heterodimers and this 6-subunit complex is metastable and conformationally flexible. Several approaches have been pursued to stabilize the Env trimer for vaccine purposes, which include structure-based design, high-throughput screening, and selection by mammalian cell display. Here, we employed directed virus evolution to improve Env trimer stability. Accordingly, we deliberately destabilized the Env gp120-gp41 interface by mutagenesis in the context of replicating HIV-1 LAI virus and virus evolution over time. We identified compensatory changes that pointed at convergent evolution, as they were largely restricted to specific Env regions, namely, the V1V2 domain of gp120 and the HR1 and HR2 domain of gp41. Specifically, S614G in V1V2 and Q567R in HR1 were frequently identified. Interestingly, the majority of the compensatory mutations were at distant locations from the original mutations and most likely strengthen intersubunit interactions. These results show how the virus can overcome Env instability and illuminate the regions that play a dominant role in Env stability.

**IMPORTANCE** A successful HIV-1 vaccine most likely requires an envelope glycoprotein (Env) component, as Env is the only viral protein on the surface of the virus and the target for neutralizing antibodies. However, HIV Env is metastable and flexible because of the weak interactions between the Env subunits, complicating the generation of recombinant mimics of native Env. Here, we used directed viral evolution to study Env stability. We deliberately destabilized the interface between Env subunits and explored the capacity of the virus to repair trimer instability by evolution. We identified compensatory mutations that converged in specific Env locations: the apex and the trimer interface. Selected mutations enhanced the stability of recombinant soluble Env trimer proteins. These results provided clues on understanding the structural mechanisms involved in Env trimer stability, which can guide future immunogen design.

## INTRODUCTION

HIV-1 infection of target cells is mediated by the envelope glycoprotein (Env) trimer (1) on the surface of the virus. The Env trimer first attaches to the CD4 receptor of the target cell, which induces a conformational change in the Env that leads to its binding to the coreceptor (CCR5 or CXCR4) and ultimately to the fusion of the Env trimer with the cell membrane (1, 2). The Env protein is the only viral protein on the surface of the virus and therefore of the only target for neutralizing antibodies (NAbs), making Env the major objective of vaccine designs with the goal of eliciting NAbs.

Env consists of three gp120 subunits that are noncovalently attached with three gp41 subunits. The interactions between the Env subunits are very weak, resulting in the ubiquitous presence of dissociated subunits that expose nonneutralizing antibody (non-NAb) epitopes, which probably leads to immune decoy (3, 4). To avoid dissociation of the individual gp120 and gp41 subunits in the context of soluble Env immunogens, we have previously designed prototype soluble Env trimers, termed SOSIP.664 trimers, which are stabilized by an I559P mutation in gp41 and by a disulfide bond between gp41 and gp120 (501C-605C) (5, 6). The clade A BG505 SOSIP.664 trimer enabled us to solve the atomic structure of the Env trimer, which was followed by structures of Env trimers from different subtypes (7–12). High-resolution structures of membrane-derived nonstabilized Env trimers showed that the SOSIP trimers resembled the native Env trimer structure (13–16). Vaccination with these soluble native-like trimers induced NAbs against the autologous neutralization-resistant (tier 2) virus isolates in rabbits and in nonhuman primates, but this NAb response needs to be improved to induce broadly neutralizing antibodies (bNAbs) able to protect against the wide variety of HIV-1 variants (12, 17–31). To increase the induction of NAbs, a number of groups have improved native-like trimer antigenicity and stability by using structure-based design, high-throughput screening, and mammalian cell display (12, 17, 21, 32–47).

Taking advantage of the enormous capacity of retroviruses to mutate and evolve, virus evolution has been used as a tool to study and optimize viral and nonviral proteins. For instance, virus evolution has helped select oncolytic viruses that can potently replicate in carcinogenic microenvironments; it has allowed the development of adeno-associated viruses with specific tissue tropisms; and it has enabled the design of an optimized tetracycline gene expression system using an HIV-1 virus that depended on the tet-on system (48–50). The evolutionary capacity of HIV-1 has also been exploited to improve HIV-1 Env immunogens (33, 51–55). For example, Bontjer et al. created Env protein mutants that lacked variable loops 1 and 2 (V1V2) (53). The resulting proteins folded and expressed poorly, but this defect could be repaired by forced virus evolution. Furthermore, Leaman et al. subjected the clade B LAI virus to several cycles of physical and chemical destabilization and used directed evolution to identify mutations that could overcome trimer instability (52). Finally, de Taeye et al. used virus evolution to repair expression defects caused by introducing a disulfide bond in the V1V2 domain (55). Thus, virus evolution is a powerful technique that can provide solutions to protein engineering problems that cannot be obtained easily by design.

Here, we explored whether and how HIV-1 can repair Env trimer instability by conducting evolution experiments with viruses in which we deliberately destabilized the gp120-gp41 interface by mutagenesis. The mutagenesis strategy was guided in part by previous studies revealing residues that were important for Env trimer stability (51, 56–65). By studying the evolved viruses, we identified compensatory mutations and found that these were localized in specific regions of Env, mostly at the trimer apex and gp41 trimer interface. These mutation hot spots reveal convergent virus evolution upon destabilization of the gp120-gp41 interface. The results will help to understand the structural mechanisms involved in stability of the functional Env trimer, which can guide future immunogen design.

## RESULTS

### Selection and design of mutations at or near the gp120-gp41 interface.

**FIG 1:**
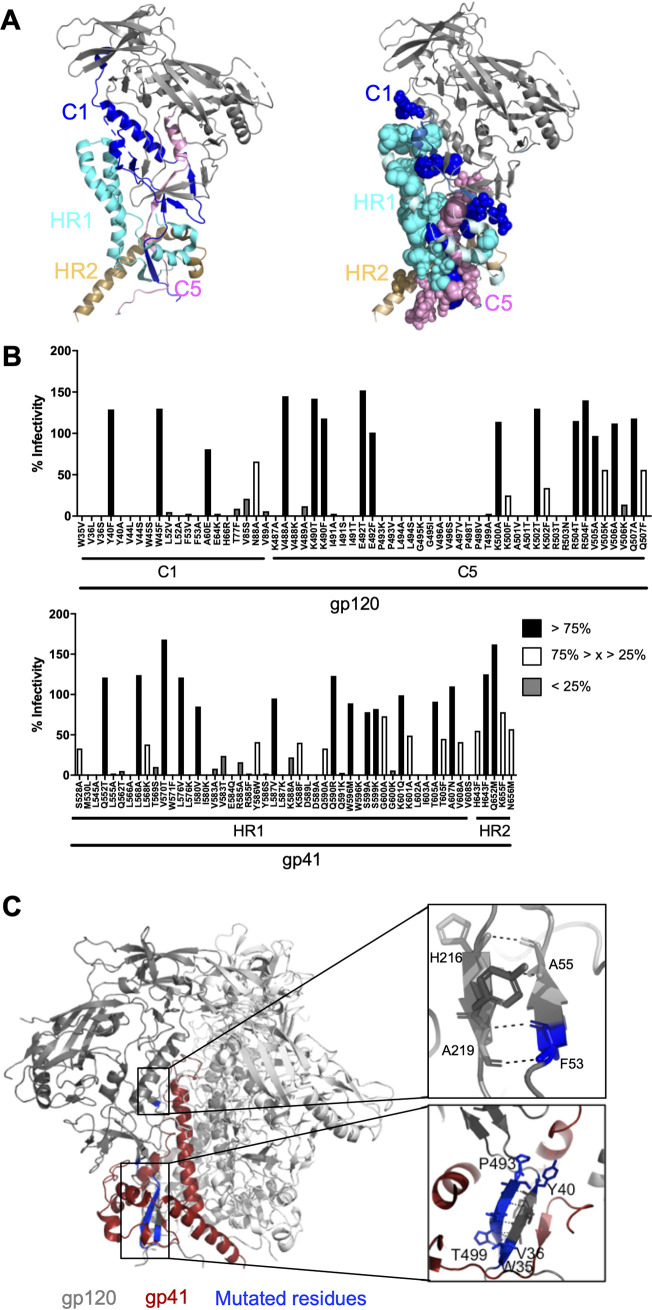
Design of mutations at and near the gp120-gp41 interface of the Env trimer. (A, left) Side view of one protomer of the membrane-derived JR-FL Env trimer (PDB entry 5FUU) (13). The structure is shown in ribbon. The color code is the following: C1 of gp120 in blue; C5 of gp120 in pink; the remaining domains of gp120 in gray; HR1 of gp41 in cyan; and HR2 of gp41 in brown. (Right) Same side view as in panel A with the mutations introduced at or near the gp120-gp41 interface, shown as spheres. The sizes of the spheres are proportional to the total number of evolution cultures that were initiated with viruses having a mutation at the respective residues (see Table 1 for further details). The mutations are colored according to the color scheme of the left panel. (B) Relative infectivity of the 72 mutant HIV-1 LAI viruses with mutations at the gp120-gp41 interface, as measured on TZM-bl reporter cells. Infectivity of the wild-type HIV-1 LAI virus was set at 100%. Mutations that had no effect on infectivity (>75%) are shown in black, mutations with intermediate effect (75% > *x* > 25%) are shown in white, and mutations that resulted in minimal infection (<25%) are in gray. (C) Side view of the membrane-derived JR-FL Env trimer (PDB entry 5FUU) (13). The gp120 of one protomer is colored in gray and gp41 of the same protomer in red. The other protomers are in white. Areas of interest in the C1 domain where mutations were introduced that resulted in lack of infectivity are boxed and enlarged in the insets on the right. The upper right panel highlights a two-stranded β-sheet consisting of residues located in C1 and C2, shown as sticks (F53-A55 and H216-A219, respectively). The lower panel highlights a two-stranded β-sheet consisting of residues located in C1 and C5, also shown as sticks (W35-Y40 and P493-T499, respectively). Residues that were mutated in this study are colored in blue (see panel B).

To investigate how single substitutions in the envelope glycoprotein complex (Env) of HIV-1 affect viral infectivity, we mutated a molecular clone of clade B LAI HIV-1 virus. Previous studies showed that interactions between the amino acids located at the gp120-gp41 interface are necessary for maintaining the integrity of the Env protein and therefore are needed for viral entry (5, 8, 10, 41, 51, 56–66) (Table 1). We selected 72 positions, located in the three regions that are at or near the trimer interface: the C1 and C5 domains in gp120 and most of the gp41 ectodomain (Fig. 1A, Table 1). To decrease the chance of reversion to the original amino acid, all the amino acid substitutions involved at least two nucleotide changes. In many cases, we also explored two different substitutions at a given position, one conservative change and one more dramatic change. For example, V36 was changed to L (conservative) and S (nonconservative), Y40 was changed to F (conservative) and A (nonconservative), and so on for many other positions. The reason to follow this strategy was that at positions that are crucial for the gp120-gp41 interaction, even a conservative change might impact virus replication and drive evolution, while a more dramatic change might have such severe consequences that evolution would be difficult or impossible. On the other hand, at a residue with a minor role in gp120-gp41 stability, a conservative change might not impact virus infectivity and replication and might not drive evolution, while a more dramatic change at such a position might be more prone to facilitate virus evolution. Indeed, both scenarios applied to our study (see below). Overall, this resulted in the production of 122 mutant viruses (see Table S1 in the supplemental material).

**TABLE 1:** Mutations introduced at or near the gp120-gp41 interface^*a*^

Region and position	Original amino acid	Mutation	No. of cultures	Reference	Mutation in reference
C1					
35	W	V	6	63	A
36	V	L/S	4	59/63	A
40	Y	F/A	6	59/63	A
44	V	L/S	6	40/63	A
45	W	S/F	4	59/63	A
52	L	V/A	6	58/63	A
53	F	V/A	6	58/63	A
60	A	E	4	57/58	G
64	E	K	4	57	K
66	H	R	4	57/58	A/R
77	T	F	2	58	A
85	V	S	4	58	A
88	N	A	5	58/59	A
89	V	A	4	40/58	A
C5					
487	K	A	4	51/58	A/E/N
488	V	A/K	4	40/60	A
489	V	A	4	60	A
491	I	A/S/T	8	40/59/60	A
492	E	T/F	4	60	A
493	P	K/V	4	59/60	A
494	L	A/S	6	60	A
495	G	K/I	4	59	A
496	V	A/S	6	60	A
497	A	V	4	60	A
498	P	V/A	5	60	A
499	T	A	4	60	A
501	A	V/T	6	5/59	K/C
502	K	T/F	4	60	A
503	R	T/N	4	60	A
504	R	T/F	4	60	A
505	V	A/K	6	60	A
506	V	A/K	4	60	A
507	Q	A/F	4	60	A
HR1					
528	S	A	4	56	T
530	M	L	4	56	S
545	L	A	2		
552	Q	T	4	56/60	L/A
555	L	A	3	56/60	G/A
562	Q	T	4	56/60	L/A
566	L	A	2	56/60	G/A
568	L	A/K	6	56/60	A
569	T	S	4	60	A
570	V	T	4	60	A
571	W	F	4	56/60	R/A
576	L	V/K	4	60	A
580	I	V/K	6	60	A
583	V	A/T	4	60	A
584	E	Q	2	56/60	A
585	R	A/F	6	60	A
586	Y	W/S	6	60	A
587	L	V/K	4	60	A
588	K	A/F	4	57/60	A/E
589	D	L/A	4	56/60	L/A
590	Q	A/R	6	57/60	A/E
591	Q	K	2	51/60	A/L
596	W	M/K	4	56	W/M
599	S	A/K	6		
600	G	A/K	6	57	A/K
601	K	Q/A	4	57	A
602	L	A	2		
603	I	A	2		
605	T	A/F	6	5	C
608	V	A/S	4	56	S
HR2					
643	H	F	6	57	F/K
652	Q	M	2	56	L
655	K	F	2		
656	N	M	2	56	L

a Mutations were chosen for evolution studies and literature references on which the selection of the mutations is based are listed. Env numbering is based on the HXB2 sequence.

### HIV-1 infectivity is reduced by mutations at or near the gp120-gp41 interface.

We quantified the capacity of the 122 mutant viruses to infect TZM-bl reporter cells compared to the wild-type (wt) HIV-1 LAI virus (set as 100%). A subset of the 122 mutations had no major effect on virus infectivity (>85% infectivity compared to wild-type virus; 24 mutations), while 38 mutations resulted in a moderate reduction of viral infectivity (15 to 85% infectivity). The majority of mutants had a dramatic impact on infectivity (<15% infectivity; 60 mutants). When we compared infectivity levels of our panel of viruses with infectivity levels or viral replication of similarly mutated viruses from previous studies, the results were generally consistent, with a few exceptions (52, 56–58, 60–65).

Mutations in the C1 and C5 regions of Env resulted, with a few exceptions, in poor or no viral infectivity (Fig. 1B). Inspection of the local structure rationalizes these results. Specifically, residues 494 to 499 in C5 form a β-strand that pairs with another β-strand composed of residues 35 to 40 in C1 through backbone and side chain hydrogen interactions to form a two-stranded β-sheet linking C1 with C5. The integrity of this β-sheet is evidently necessary for Env function (Fig. 1C). Similarly, amino acids F53-A55 in C1 form a β-sheet with amino acids H216-A219 in C2 that appears important for Env function (Fig. 1C). Two conservative substitutions, Y40F and W45F, in C1 did not impair viral infectivity, probably because the introduced amino acids have properties intrinsically similar to the original amino acid and are not expected to disrupt the local secondary and tertiary structure (Fig. 1B). In contrast to the gp120 mutants, where lack of infectivity corresponded to specific regions of Env, poor viral infectivity of the gp41 mutants was observed along the entire region (Fig. 1B).

### Mutation of the gp120-gp41 interface triggers convergent evolution.

To assess how HIV-1 could accommodate the introduced mutations, all 122 viruses were passaged for a prolonged time in independent evolution cultures. Most mutants were evaluated in duplicate cultures, but some mutants were tested in triplicate or quadruplicate, amounting to a total of 249 independent virus cultures. The cultures were maintained for at least 8 weeks and up to 24 weeks, until they reached an apparent replication capacity similar to that of the wild-type virus, as judged by the formation of syncytia and depletion of target cells (see Table S1 in the supplemental material). We observed three classes of replication-evolution patterns. First, in ∼26% of the cultures (64 of 249), virus replication was apparent from the initiation of the culture. Second, in ∼44% of the cultures (107 of 249), no rapid virus spread was observed initially, but over time the virus started to spread more rapidly, which we took as evidence for virus evolution. Third, in ∼31% of the cultures (78 of 249), we did not observe any replication over a 3-month time frame, after which the cultures were stopped. The viruses in these cultures correspond to those that displayed the lowest infectivity in the TZM-bl assays (Fig. 1B, Table S1).

The proviral DNA from the replicating viruses was subsequently isolated and sequenced every 4 weeks of culture. Among the viruses that recovered replication capacity, 10% had reverted the mutated amino acid to the wild-type residue (first-site reversion). As pointed out above, we always mutated two nucleotides, but the degeneracy of the codon allowed the generation of wild-type revertants (at the amino acid level) by changing only one nucleotide (Table S1).

In the other 90% of the cultures the virus gained replication capacity by introducing compensatory mutations at different positions (second-site reversion). Interestingly, 89% of these compensatory mutations were found in other HIV-1 isolates in the Los Alamos database (www.hivmut.org/lanl), suggesting that the virus has limited choices when incorporating compensatory mutations. However, 11% of the reversions were never found in sequences of the natural isolates present in the Los Alamos database, suggesting that in these cases very unusual amino acids were selected to solve the problems introduced by the original mutation.

**FIG 2:**
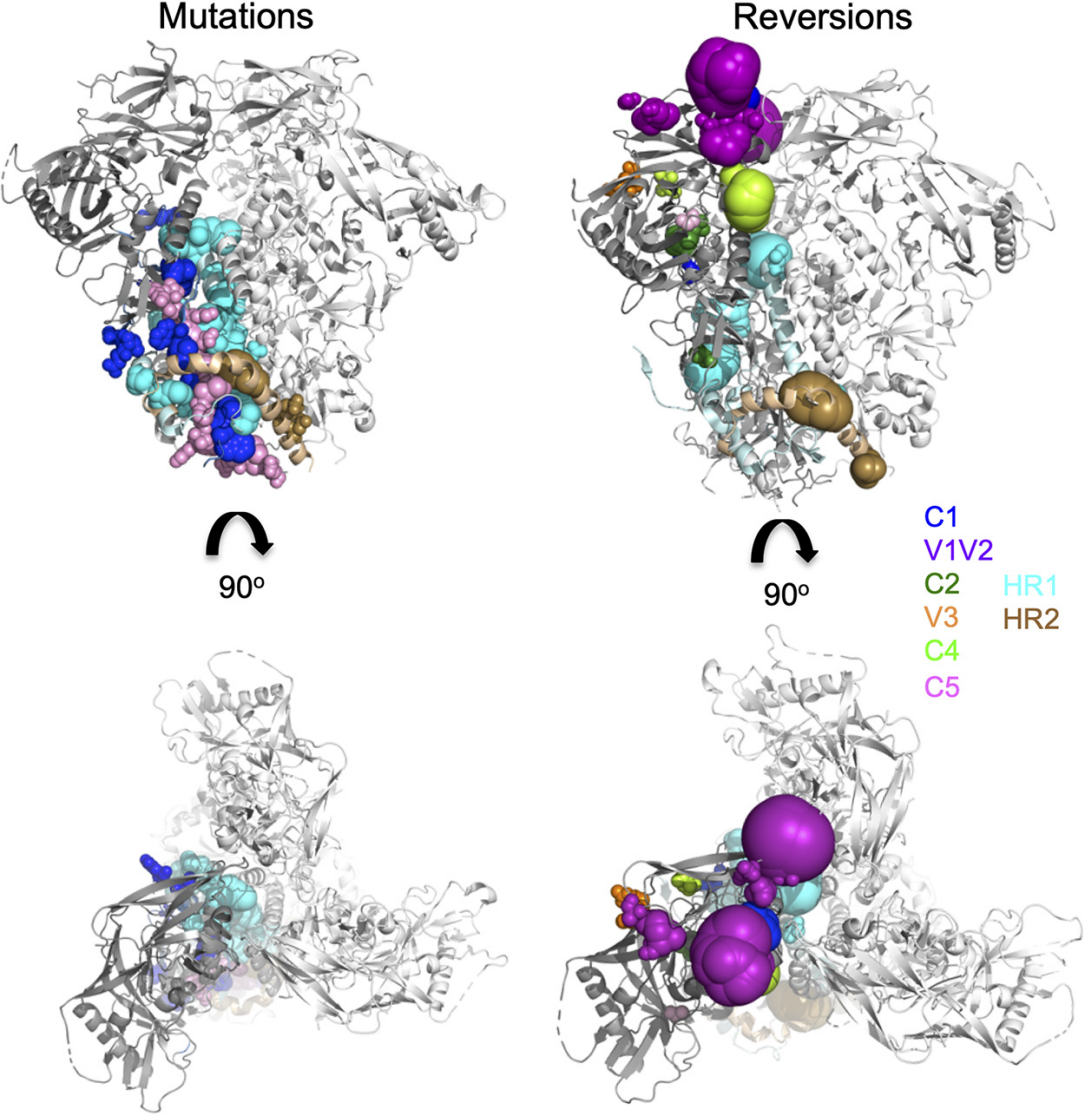
Location and frequency of original mutations and compensatory mutations acquired after evolution. The left panels show side (upper) and top (lower) views of the membrane-derived JR-FL trimer (PDB entry 5FUU) (13). One protomer is shown in the same color code as that in the left panel. The other two protomers are shown in white. The locations of mutations that were introduced at or near the gp120-gp41 interface are colored in one protomer as in Fig. 1A. The right panels show the locations of compensatory mutations that occurred over prolonged evolution in at least two cultures, irrespective of the original mutations present in the viruses in these cultures, plotted as spheres. The sizes of the spheres are proportional to the number of evolution cultures in which reversions at the respective residues were present. The compensatory mutations are colored based on the region in which they are located: C1 in blue; V1V2 in magenta; C2 in dark green; V3 in orange; C4 in green; C5 in pink; HR1 in cyan; and HR2 in brown.

To understand the molecular mechanisms that the virus adopted to cope with the introduction of destabilizing mutations, we inspected the regions of the Env trimer where the compensatory mutations appeared (Table 2). We plotted the compensatory mutations that occurred in more than one culture on the structure of the membrane associated HIV-1 Env JR-FL trimer (PDB entry 5FUU) (13), sized by its frequency of occurrence in the 249 evolution cultures and colored per domain (Fig. 2A, Table 2). Surprisingly, we observed that the evolution pathways of the mutated viruses converged by introducing compensatory mutations at specific locations distant from the original site of mutation, predominantly the V1V2 loop. Reversions were also frequently found at the gp120-gp41 interface but usually distant from the site of mutation (Fig. 2A).

**TABLE 2:** Location of the compensatory mutations and frequency of occurrence in viral cultures^*a*^

Region and position	Original amino acid	Mutation	No. of cultures
C1			
60	A	K/T	2
128	S	R	5
V1V2			
139	N	S	2
143	S	G	2
145	G	E/I	4
146	E	K/S	2
160	N	K	3
162	S	R	2
163	T	K	2
164	S	G	15
170	Q	R	2
181	I	L	2
185	D	N/G	8
188	T	A/N	8
190	S	R	9
192	T	K	3
194	T	A/K	5
C2			
244	T	A	2
254	V	I/A	3
255	V	I	2
V3			
297	T	A	2
298	R	K	2
C4			
429	E	K	6
433	A	T	4
439	I	V	2
C5			
471	G	R	2
HR1			
550	Q	H	6
557	R	K	3
558	A	T	2
560	E	G	3
567	Q	R	7
570	V	A	2
595	I	M	3
607	A	S	3
HR2			
643	H	Y	6
656	N	S	4

a The compensatory mutations that occurred in more than one culture are listed.

Many mutations (42%) emerged in V1V2 of gp120, which is a very flexible region of the gp120 Env subunit that only contains ∼43% conserved residues among different HIV subtypes (www.hivmut.org/lanl). Structures of soluble native-like BG505 Env trimers showed that the V1V2 domains from the three protomers form the “cap” of the trimer at the apex, which is important for stabilizing the trimer interface (10, 16, 44, 67–72). Compensatory mutations in the V1V2 region might increase trimer stability by making noncovalent interactions with V3 underneath or at the trimer apex, within and between protomers (9, 13, 41, 73). The most common compensatory mutation, S164G, located at the V2 loop, was found in 21% of the cultures. Since amino acid 164 is situated near the adjacent protomer, its substitution may stabilize the trimer by increasing the noncovalent interactions at the trimer apex with the other protomer (residues 195 to 198) (72).

In contrast to V1V2, the core of gp120 is relatively rigid, and only 12% of the total reversions were found in this region. Interestingly, we found mutations in C4, specifically E429K, A433T, and I439V. This region corresponds to a loop that interacts with the β-strand in C2, which expands from residue S199 to Q203. Stabilization of this part of the viral Env is important to prevent transitioning from closed (prefusion) to open (receptor-bound) conformations (44, 70, 74). Kwon et al. and Guenaga et al. introduced point mutations (E429R and K432Q) or a disulfide bond (201C-433C) in this same loop to stabilize the core of the trimer (44, 75). In line with this observation, our study suggests that the virus can use a similar strategy to compensate for destabilization at the gp120-gp41 interface.

HR1 in gp41 is a region of the Env trimer located near the gp120 subunit that changes conformation when Env transitions from prefusion to postfusion states (2, 76). According to the high b factor of this region, measured by X-ray crystallography, HR1 is a relatively flexible region in full-length and SOSIP.664 Env trimers (9, 10, 13, 67). Thirteen percent of the cultures showed compensatory mutations at this region, between positions 557 and 570. The compensatory mutation at Q567R was previously shown to enhance stability of KNH1144 SOSIP trimers compared to JR-FL SOSIP trimers and also to induce resistance to the HIV-1 fusion inhibitor T2635 (57, 77). Our study corroborates the important role of this residue in stabilizing the prefusion state of the Env trimer.

Finally, we detected 8% of mutations in the HR2 region of the Env trimer, mostly at residues H643Y and N656S. These two residues have been previously implicated in membrane fusion and gp120-gp41 dissociation, respectively (56, 65). According to cryoelectron microscopy (cryo-EM) and crystal structures of the Env protein, these residues might be involved in intersubunit and interprotomer interactions, respectively, i.e., interactions with residues L493 in the C5 from the same protomer and with residues M535 in HR2 from the adjacent protomer (10, 13, 41, 73).

To better comprehend how the virus copes with destabilization of the Env interface, we examined the regions of the Env trimer where the destabilizing mutations were introduced together with the regions where the compensatory mutations appeared. Mutations introduced in C1 and C5 of gp120 induced compensatory mutations in both gp120 and gp41 subunits of the Env, specifically the V1V2 loop of gp120 and HR1 and HR2 of gp41 (Table 2). Conversely, mutations introduced in gp41 generally resulted in compensatory mutations in the V1V2 loop (Table 2). Overall, we observed that the compensatory mutations were mostly located far from the original destabilizing mutations (Table 2), suggesting that destabilization of the gp120-gp41 interface can be compensated for by strengthening the intersubunit interactions elsewhere (see Fig. 5).

### Substitutions S164G and Q567R stabilize mutant virus-associated Env.

To analyze the effect of selected compensatory mutations identified above on virus infectivity and Env stability, we chose two compensatory mutations that appeared in multiple cultures and that were located in different regions of the Env trimer (S164G in V2 and Q567R in HR1). Next, we constructed molecular clones that contained the original mutation, the compensatory mutation, or both, and we assessed virus infectivity of TZM-bl reporter cells.

**FIG 3:**
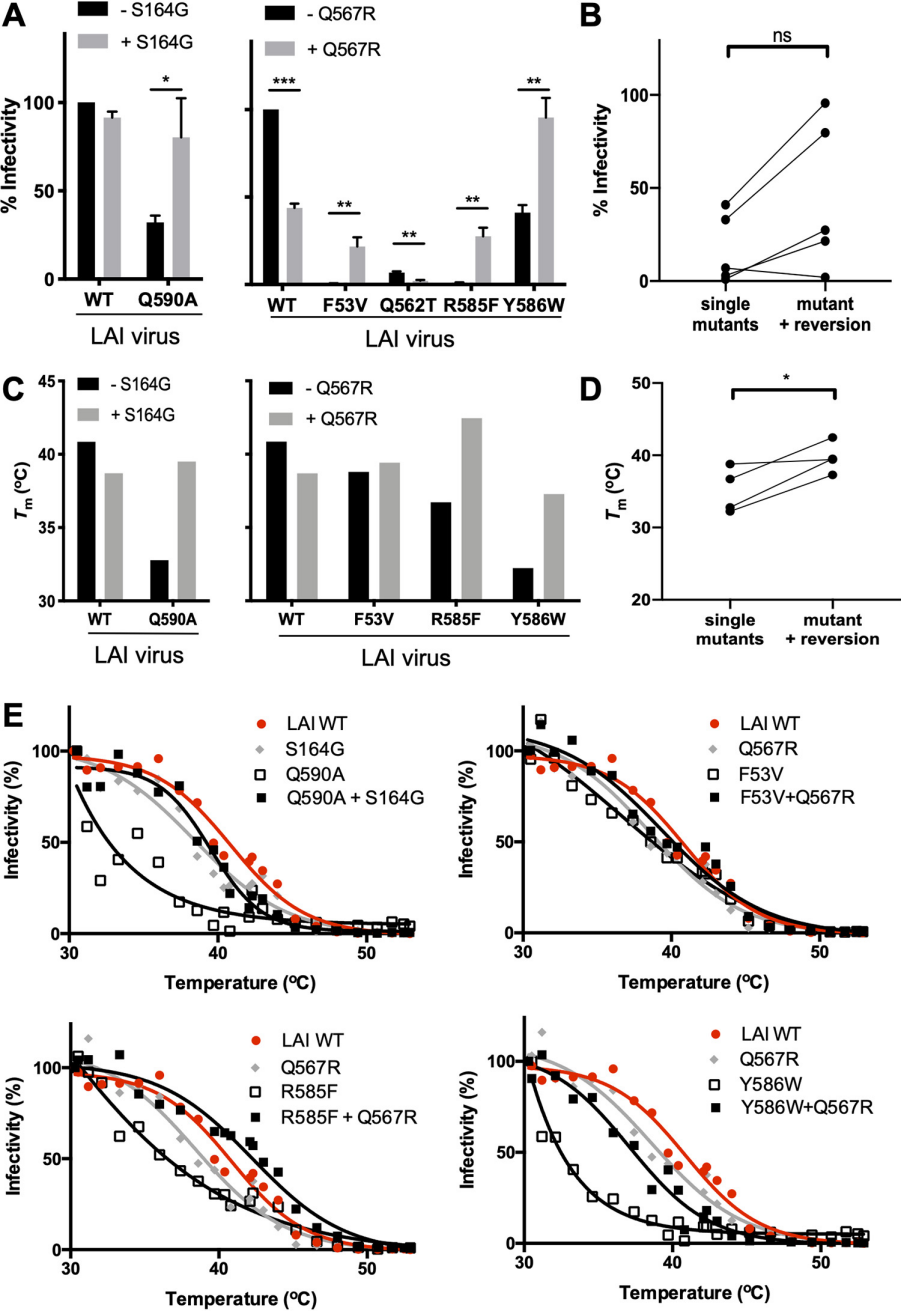
Compensatory effects of reversions on virus infectivity and stability. (A) Infectivity of the wild-type HIV-1 LAI and mutated viruses, carrying (gray bars) or not carrying (black bars) the compensatory mutations S164G (left) or Q567R (right), as measured on TZM-bl reporter cells. Infectivity of the wild-type HIV-1 LAI virus was set at 100%. (B) Paired comparison (paired *t* test) of viral infectivity between mutant viruses with or without the compensatory mutation S164G or Q567R. (C) The midpoints of thermal denaturation (*T_m_*) of the wild-type virus and the mutant viruses, carrying (gray bars) or not carrying (black bars) the compensatory mutation S164G (left) or Q567R (right), calculated using the data shown in Fig. 4E. (D) Paired comparison of *T_m_* values of viruses with and without the compensatory mutation S164G or Q567R. (E) Thermostability of the wild type (in red), viruses containing only the S164G or Q567R compensatory mutation (in gray), mutant viruses (in black, open symbols), and mutant viruses that also contained the compensatory mutation S164G or Q567R (in black, closed symbols). The viruses were incubated at a range of temperatures for 1 h, and their residual infectivity was determined.

When we introduced the S164G mutation in the wild-type LAI virus, we observed that it had no effect on virus infectivity. The S164G mutation was found in many different mutant viruses, one of which had the Q590A substitution. Therefore, we introduced the S164G mutation in the context of a mutant virus that contained the Q590A substitution. We observed that the Q590A mutant virus showed decreased infectivity (33% compared to the wild type; *P* = 0.0003) (Fig. 3A), while the introduction of the S164G mutation recovered the infectivity levels to 80% (*P* = 0.0145) (Fig. 3A).

Next, we introduced the Q567R mutation in the wild-type virus and observed a reduction of infectivity (44% infectivity compared to the wild type; *P* < 0.001). We selected five mutant viruses that acquired the Q567R reversion (W35V, F53V, Q562T, R585F, and Y586W), and we assessed the infectivity of these mutants compared to double mutants that also contained Q567R. The introduction of Q567R to the viruses containing F53V, R585F, and Y586W resulted in partial recovery of viral infectivity (from 3% to 22%, *P* = 0.04, from 1% to 27%, *P* = 0.0015, and from 41% to 96%, *P* = 0.0016, respectively) (Fig. 3A). Overall, addition of Q567R and S164G to the mutant viruses recovered infectivity, except when Q567R was introduced in the virus containing the Q562T mutation (from 7% to 2%, *P* = 0.0021) (Fig. 3A and B).

To provide a mechanistic explanation for the compensatory effects of the S164G and Q567R substitutions, we evaluated the thermostability of wild-type LAI virus as well as single and double mutants. We exposed the viruses for 1 h to escalating temperatures and assessed the remaining infectivity on TZM-bl cells (52, 64). In this assay, the wild-type LAI virus has a midpoint to thermal denaturation (*T_m_*) of ∼40.9°C, which is similar to previous results (52, 64). The viruses containing only the compensatory mutation S164G or Q567R showed subtly lower *T_m_* values (∼39.1°C and 38.8°C, respectively) (Fig. 3C and E).

Destabilizing effects were clearly discernible for the viruses that contained the R585F, Y586W, or Q590A substitution (*T_m_* values of ∼36.7°C, ∼32.2°C, and ∼32.8°C, respectively), consistent with their reported effects on viral infectivity or escape from T2635 fusion inhibitor (60, 64) (Fig. 3C and E). The destabilizing effect of Q590A (Fig. 3C and E) was substantially more pronounced than the effect of Q590E (64). In contrast, introduction of the F53V mutation only decreased the thermostability of Env slightly (*T_m_* value of ∼38.7°C) (Fig. 3C and E).

Introduction of the S164G mutation into the Q590A background dramatically increased the thermostability, as shown by an increase of the *T_m_* from ∼32.8°C to ∼39.5°C, a value close to that of wild-type virus (Fig. 3C and E). The incorporation of the Q567R mutation restored the stability of virus mutants containing the R585F and Y586W mutations, as shown by increases in *T_m_* values from ∼36.7°C (R585F) to ∼42.3°C (R585F+Q567R) and from ∼32.2°C (Y586W) to ∼37.3°C (Y586W+Q567R) (Fig. 3C and E). In contrast, the Q567R mutation did not notably increase the stability of the F53V mutant (*T_m_* values of ∼39.4°C for the double mutant versus ∼38.7°C for the F53V single mutant). Collectively, these data indicate that deliberate destabilization of the Env trimer by mutation of the gp120-gp41 interface can be overcome by virus evolution by incorporating compensatory mutations such as S164G and Q567R that restore the stability of the Env protein (*P* = 0.0437) (Fig. 3D).

### Substitutions S164G and Q567R stabilize mutant SOSIP trimers.

Next, we analyzed the effect of the compensatory mutations studied above on soluble Env trimers. Accordingly, we produced LAI SOSIP.664 trimers that contained the original mutation, the compensatory mutation, or both, and we assessed Env stability and antigenicity.

**FIG 4:**
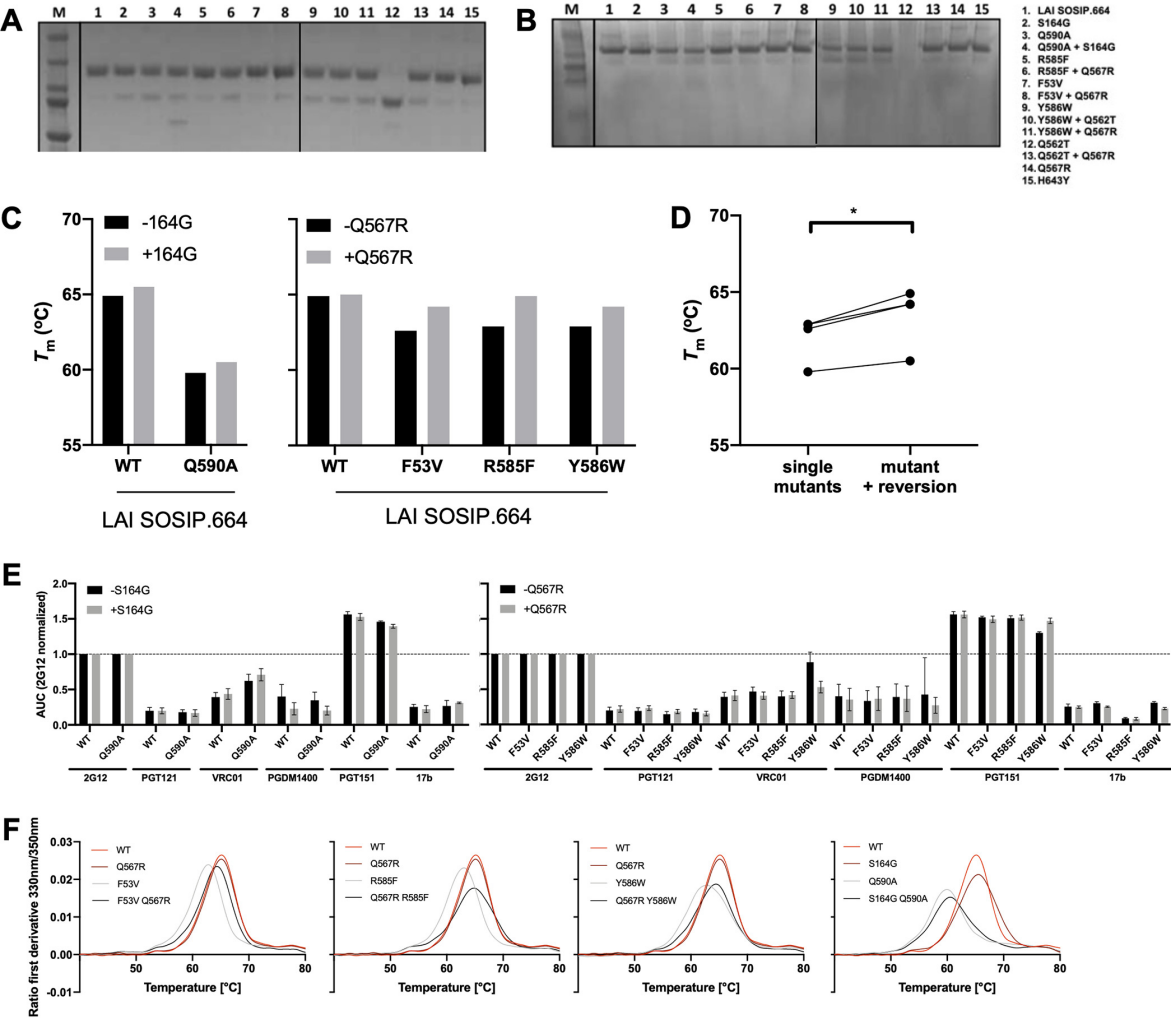
Biochemical, biophysical, and antigenic characterization of wild-type LAI virus and SOSIP.664 mutants. (A) Reducing SDS-PAGE was used to determine cleavage efficiency. (B) BN-PAGE was used to determine trimerization of the SOSIP.664 proteins. (C) The midpoints of thermal denaturation (*T_m_*) of LAI SOSIP.664 (WT) and the mutant SOSIP.664 Env proteins, carrying (gray bars) or not carrying (black bars) the compensatory mutations S164G and Q567R. The data were calculated using the data shown in panel F. (D) Paired comparison of *T_m_* values of mutant LAI SOSIP.664 with and without the compensatory mutations S164G or Q567R. (E) Antigenicity was assessed by BLI. Five broadly neutralizing antibodies and one nonneutralizing antibody were tested (2G12, PGT121, VRC01, PGDM1400, PGT151, and 17b). Bars represent the area under the curve normalized to 2G12 binding. (F) Thermostability of LAI SOSIP.664 and mutants was assessed with differential scanning fluorimetry (DSF). The first derivative of the curves acquired with DSF is shown.

The different variants of LAI SOSIP.664 trimers were expressed in HEK 293F cells and purified via PGT151-affinity chromatography, as previously described (45). All the Env proteins were fully cleaved and homogeneous, as assessed by reducing and nonreducing SDS-PAGE (Fig. 4A). Native-PAGE analysis showed that the SOSIP.664 mutants migrated mostly as trimers, except for the proteins that contained the Y586W mutation, which showed a stronger dimer band, indicating that the trimer is more likely to fall apart (Fig. 4B). Next, we assessed the ability of the LAI Env proteins to bind broadly neutralizing antibodies, and we observed that all LAI SOSIP.664 mutants retained the ability to bind the broadly neutralizing antibodies 2G12, PGT121, PGDM1400, PGT151, and VRC01 and not the nonneutralizing antibody 17b (Fig. 4E).

Next, we assessed the trimer thermostability by differential scanning fluorimetry (DSF). When we introduced the S164G mutation in the wild-type LAI SOSIP.664 Env protein, we observed that the thermostability of the trimer increased by 0.6°C (Fig. 4C). Since this mutation was found in viruses that contained the Q590A substitution, we introduced the Q590A substitution in LAI SOSIP.664 and in the context of the mutant SOSIP.664 that contained S164G. We observed that the Q590A SOSIP.664 mutant showed decreased thermostability (*T_m_* value of 59.8°C) compared to the wild-type SOSIP.664 (*T_m_* value of 64.9°C) (Fig. 4C and F), similar to the decrease in infectivity observed when the thermostability of Q590A mutant LAI virus was assessed (Fig. 3A, C, and E). The introduction of the S164G mutation in SOSIP.664 that contained the Q590A mutations increased its thermostability by a modest 0.7°C, similar to the effect of the S164G substitution on the wild-type SOSIP.664 protein (Fig. 4C and F).

The incorporation of the S164G and Q567R mutations into wild-type SOSIP.664 restored the stability of the Env proteins containing the F53V, R585F, Y586W, and Q590A mutations (Fig. 4D) (*P* = 0.0145), as shown by increases in *T_m_* values from 62.6°C (F53V) to 64.2°C (F53V + Q567R), from 62.9°C (R585F) to 64.9°C (R585F + Q567R), and from 62.9°C (Y586W) to 64.2°C (Y586W + Q567R) (Fig. 4C and D). In conclusion, destabilization of the Env trimer results in the appearance of compensatory mutations that increase the infectivity of the virus and stability of the Env protein.

## DISCUSSION

**FIG 5:**
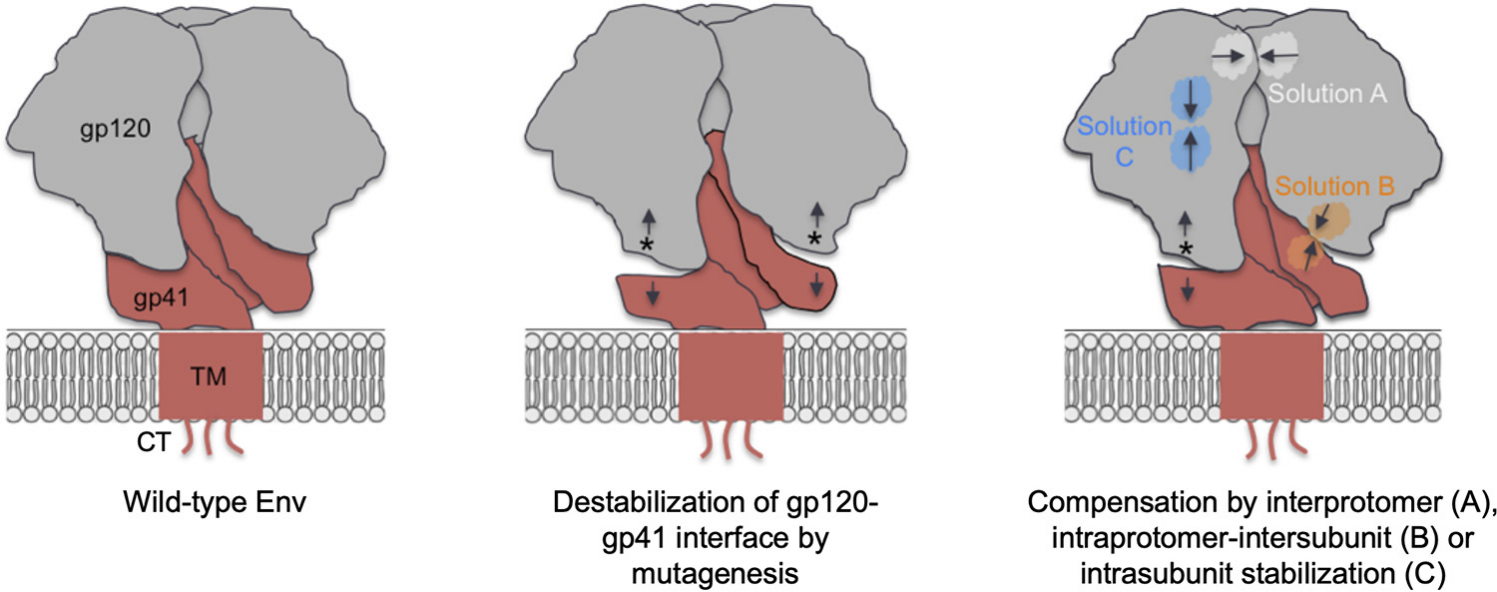
Viral solutions to compensate for destabilization of the gp120-gp41 interface. (Left) Cartoon representation of the wild-type HIV-1 LAI Env trimer. The gp120 subunits are shown in gray, and the gp41 subunits, including the transmembrane domain (TM) and the cytoplasmic tail (CT), are shown in red. (Middle) Introduction of a mutation (shown as an asterisk) at or near the gp120-gp41 interface destabilizes the interface (shown by less intimate contacts). (Right) Viral evolution introduces compensatory mutations that restore Env stability. The virus employs three solutions to compensate for the destabilization of the gp120-gp41 interface. Solution A involves the stabilization of interprotomer interface (between gp120 subunits [as shown], between gp41 subunits, or between gp120 from one subunit and gp41 from another subunit); solution B involves the stabilization of the intraprotomer intersubunit gp120-gp41 interfaces (but distal from the site of mutation); and solution C involves the stabilization of intrasubunit interactions.

We describe how the HIV-1 LAI virus can overcome the destabilizing effect of mutations at or near the gp120-gp41 interface. We observed that such evolution converged, as compensatory mutations were concentrated in specific regions of the Env glycoprotein, mostly in V1V2 of gp120 and HR1 and HR2 of gp41. Overall, we observed that the virus used three strategies to compensate for the mutations that were introduced at or near the gp120-gp41 Env interface. One strategy was to stabilize the interactions between different gp140 protomers, specifically between the three V1V2 domains in gp120 or between the three HR2 domains in gp41 (solution A) (Fig. 5). The second was to stabilize the gp120-gp41 interface of the same protomer (solution B) (Fig. 5). The third was to stabilize the interaction within gp120 of one protomer (solution C) (Fig. 5). In all scenarios, the compensatory changes were usually introduced in regions distal from the original mutation (Fig. 5).

Most of the compensatory changes that we identified were found in natural HIV-1 isolates in the Los Alamos database, suggesting that the virus has a finite number of options to restore its replicating capacity. In very few rare cases, natural HIV-1 isolates contained the same mutation-reversion pairs that we observed in this study, such as V89A plus H643Y and Q507F plus E429K, suggesting that natural coevolution yielded the same solutions as our *in vitro* evolution experiments. However, we also identified a few compensatory changes that were never found in natural isolates, such as E560G and S364P.

Prior studies aimed at stabilizing the soluble HIV-1 Env trimer by using structure-based design or selection by mammalian cell display. Several research groups stabilized soluble Env trimers by introducing substitutions in the same regions, sometimes at the same amino acids, as those highlighted in our evolution studies. Introduction of point mutations in V1V2 (A154M, F156S, S164E, I165L, Q171K, G172V, Y177W, and I192R) resulted in higher trimer stability (42, 46, 47, 78). These substitutions are at the same location (S164E) or very close to the S164G change that was dominant in our evolution cultures. As an aside, we further note that the V1V2 domain is a very versatile domain that exerts multiple roles, including controlling Env stability as we show here but also in evolution from antibody escape due to its high variability (53, 79, 80).

Second, Kwon et al. (44) and Guenaga et al. (42) introduced a disulfide bond (A201C-I433C) or point mutations (E429R and K432Q) to stabilize the core of the trimer by avoiding sampling of alternative conformations. Similarly, in six cultures we identified the E429K substitution, which might stabilize the Env trimer through a related mechanism.

Third, several studies showed that stabilizing mutations or disulfide bonds can be introduced into the HR1 region of gp41 (N553S, G588R, L568D, A561P, V570H, H72C-H564C, or A73C-A561C) (32, 34, 42, 47). During our virus evolution studies, some of the destabilized viruses picked up compensatory changes in the HR1 region (R557K, A558T, E560G, Q567R, and V570A).

Finally, we observed that two compensatory mutations (S164G and Q567R) restored the stability of the soluble trimers that contained an original mutation at the interface between gp120 and gp41.

Overall, our study reveals allosteric networks for Env stability and identifies compensatory mutations found during virus evolution that could be used to stabilize soluble Env immunogens.

## MATERIALS AND METHODS

### Construction of HIV-1 LAI molecular clones.

The full-length molecular clone of HIV-1 LAI (pLAI) (81) was used to produce wild-type and mutant viruses. A total of 72 residues were mutated. The specific substitutions were partly chosen according to those used in previous studies, but we also designed substitutions with different chemical properties (51, 56–65). A subset of substitutions were located near residues A501 in gp120 (residues V496, A497, P498, T499, K500, A501, K502, R503, R504, V505, V506, and Q507) and T605 in gp41 (residues S599, G600, K601, L602, I603, T605, A607, and V608), where a disulfide bond between A501C and T605C was previously introduced successfully to stabilize soluble Env trimers (5, 7). The remaining substitutions were located in C1 (residues W35, V36, Y40, V44, W45, L52, F53, A60, E64, H66, T77, V85, N88, and V89), the C-terminal part of C5 (residues K487, V488, V489, K490, I491, E492, P493, L494, and G495) in gp120, and at various positions in gp41: in the fusion peptide (S528 and M530), in HR1 (Q552, L566, L568, T569, V570, W571, L576, I580, V583, E584, R585, Y586, L587, K588, D589, Q590, and Q591), and in HR2 (H643, Q652, K655, and N656). The plasmid pRS1 was used to introduce mutations (at least 2 nucleotide differences compared to the original nucleotide sequence in order to decrease reversions to the original amino acid) as described previously (65, 81), and the complete *env* genes were verified by DNA sequencing. Mutant *env* genes in pRS1 were cloned back into pLAI as SalI-BamHI fragments. Each virus variant was transiently transfected in 293T cells using Lipofectamine (ThermoFisher). The virus-containing supernatant was harvested 3 days posttransfection, filtered, and stored at −80°C, and the virus concentration was quantified by capsid CA-p24 enzyme-linked immunosorbent assay (ELISA) (57). All virus mutants were produced with similar efficiency and CA-p24 levels.

### Virus infectivity determination.

The TZM-bl reporter cell line (82, 83) stably expresses high levels of CD4, CCR5, and CXCR4 and contains the luciferase and β-galactosidase genes under the control of the HIV-1 long-terminal-repeat promoter and was obtained through the NIH AIDS Research and Reference Reagent Program, Division of AIDS, NIAID, NIH (J. C. Kappes, X. Wu, and Tranzyme Inc., Durham, NC). One day prior to infection, 17 × 10^3^ TZM-bl cells per well were plated on a 96-well plate in Dulbecco's modified Eagle's medium containing 10% fetal bovine serum, 1× minimum essential medium nonessential amino acids, and penicillin-streptomycin (both at 100 U/ml) and incubated at 37°C with 5% CO_2_. A fixed amount of virus (100 pg and 500 pg CA-p24) in quadruplicate was added to the cells in the presence of 400 nM protease inhibitor saquinavir to block secondary rounds of infection (Roche, Mannheim, Germany) and 40 μg/ml DEAE in a total volume of 200 μl. Two days postinfection, the medium was removed and cells were washed once with phosphate-buffered saline (PBS; 50 mM sodium phosphate, pH 7.0, 150 mM NaCl) and lysed in reporter lysis buffer (Promega, Madison, WI). Luciferase activity was measured using a luciferase assay kit (Promega, Madison, WI) and a GloMax luminometer according to the manufacturer's instructions (Turner BioSystems, Sunnyvale, CA). The relative infectivity of mutants was established by measuring their luciferase activities on TZM-bl cells, subtracting the background luciferase activity, and setting the luciferase of wild-type HIV-1 LAI at 100% (57).

### Selection of HIV-1 restoration mutations.

SupT1 cells were transfected with 1 μg DNA of either the wild-type (wt) HIV-1 LAI molecular clone or mutants in the interface region, using electroporation. Cultures were split twice weekly and, when HIV-induced cytopathic effects and/or increased CA-p24 production were apparent, virus-containing supernatant was passaged cell free onto uninfected SupT1 cells. Per mutant, 2 to 6 independent virus cultures were started. Viruses were cultured for a maximum of 6 months. Cells and supernatant samples were taken at regular time points and stored at −80°C. Cell culturing, transfections, and CA-p24 determination were performed as previously described. When the virus cultures were stopped, DNA was extracted from infected cells using the QIAamp DNA minikit (Qiagen, Valencia, CA), and the complete proviral *env* gene was PCR amplified and sequenced as previously described (51, 53, 79).

### Virus thermostability.

HIV-1 LAI and mutant viruses were incubated for 1 h at a range of temperatures (from 30°C to 67°C). Viral infectivity at each temperature was tested as described above, and the midpoint thermal denaturation (*T_m_*) was calculated after fitting nonlinear regression curves using GraphPad Prism software, version 6.0.

### Design of LAI SOSIP.664 trimers.

LAI SOSIP.664 was generated by following previously described methods (7). In short, A501C and T605C mutations were introduced to form a disulfide bond between the two subunits of the trimer, gp120 and gp41; the original furin cleavage site (REKR) was replaced by RRRRRR to enhance cleavage efficiency; the I559P mutation was introduced to stabilize the gp41 subunit of the trimer, and a stop codon was introduced at position 664 for production of soluble protein. Site-directed mutagenesis was used to generate the mutants with point mutations (QuikChange kit; Agilent, Stratagene). The presence of the mutations was verified by sequencing.

### Protein production and purification.

LAI SOSIP.664 trimers were transiently expressed in the presence of excess Furin in suspension of HEK 293F cells and purified using PGT151-affinity chromatography columns as previously described (45). Protein concentrations were determined using absorbance at UV_280_. LAI SOSIP.664 trimers were analyzed by blue native and SDS-PAGE followed by Coomassie blue dye staining as previously described (12).

### Biolayer interferometry (BLI).

Antibody binding studies were performed using an Octet K2 instrument (ForteBio). The assays were performed at 1,000 rpm in kinetics buffer (PBS supplemented with 0.1% [wt/vol] bovine serum albumin and 0.02% [vol/vol] Tween 20 at 30°C). Monoclonal antibodies were diluted in kinetics buffer (2 μg/ml) and loaded onto Prot A sensors to a final interference pattern shift of 1 nm. The sensors were equilibrated with kinetics buffer for 60 s to remove the antibody partially bound to the probe. The Env proteins, at a final concentration of 100 nM, were allowed to bind for 300 s and to dissociate for 300 s. Data analysis was performed using Octet software and GraphPad Prism.

### Differential scanning fluorometry (DSF).

Trimer stability was determined using Nano-DSF (Prometheus). Proteins were diluted to a final concentration of 1 mg/ml and loaded to the grade capillaries. The intrinsic fluorescence signal was assessed at a scan rate of 1°C/min with an excitation power of 40%. The onset and midpoint of thermal denaturation of the proteins were calculated using Prometheus NT software.
